# Acknowledgement to Reviewers of *Biomolecules* in 2013

**DOI:** 10.3390/biom4010289

**Published:** 2014-03-04

**Authors:** Biomolecules Editorial Office

**Affiliations:** MDPI AG, Klybeckstrasse 64, CH-4057 Basel, Switzerland

The editors of *Biomolecules* would like to express their sincere gratitude to the following reviewers for assessing manuscripts in 2013:
Abe, TomoakiGruber, Andreas R.Nyberg, Scott L.Akhtar, KalimGupta, VineetOgawa, YuyaAlberti, SimonHagedoorn, Peter-LeonOhtake, HisaoAn-Yuan, GuoHanefeld, UlfOlivera, AnaAndrieu-Abadie, NathalieHasunuma, TomohisaOlmo, M. DelBahadur, PratapHelbert, WilliamOnda, YayoiBai, FanHernaiz, María J.Palmans, Anja R. A.Bakunina, IrinaHorovitz, AmnonPermyakov, EugeneBaldessari, AliciaHuwiler, AndreaPichová, IvaBaumgartner, AdiImaoka, SusumuPollegioni, LoredanoBechini, AlessioIourov, IvanPottecher, JulienBeckham, Thomas H.Jaeger, HenryPrasad, KamaleshBeke-Somfai, TamásJiang, XiaoxuPruijn, Ger J. M.Benetatos, PanayotisJiménez-González, ConcepciónPutnam, Charles W.Benham, Adam M.Jin, MinghaoRamisetty, Sreenivasa RaoBerhow, MarkJohnson, PhilipReetz, Manfred T.Billich, AndreasKamiya, NorihoResch, AlissaBlinov, NikolayKannanagattu, PrasanthRichard, John P.Bouvet, MichaelKeillor, JeffreyRios, LeonardoBracher, AndreasKholodov, YaroslavRömer, WinfriedBrindley, DavidKim, Byung-geeRosa, AngeloBrockman, Mark A.Kim, RogerRudyk, OlenaBruni, PaolaKolkata, IiserSaba, Julie D.Cai, MiKondo, RyuichiroSaraiva, Maria JoaoCantone, SaraKonvalinka, JanSchmeck, BerndCarbonetti, NicholasKordula, TomaszSchmid, ThomasCarmichael, Gordon GKourist, RobertSchug, AlexanderCarty, Michael P.Krobath, HeinrichSharp, JoshChamberlain, StormyKroy, KlausSong, JinhuaChen, ChongyiKwawabata, TakeshiStamatis, HaralambosChou, KuochenLairson, L.L.Stephanie, HuangCieplak, MarekLamb, DavidSue, Shih-CheCoy, PilarLaSalle, JanineSupuran, Claudiu TCrane, Edward J.Lavandera, IvanSwenson, ErikCui, QinghuaLebrilla, Carlito BTakabe, KazuakiCurino, Alejandro C.Lee, Chang-SooTavan, PaulDalmay, TamasLi, JingjingTenkanen, MaijaDas, KalyanLi, MinThomas, Christophe M.De Carvalho, CarlaLiberles, DavidTortorella, DomenicoDe Vries, Ronald P.Lin, PengTuroverov, KonstantinDevivo, MarcoLitvinov, RustemUversky, VladimirDykes, CarrieLopez Garcia, IsabelVas, MariaEako, HiroshiLouis, J.Virnau, PeterElez-Martínez, PedroLutz, StefanVolkman., B. F.Fabrias, GemmaMahajan, R. K.Waheed, AbdulFaísca, PatríciaManfredi, Kirk P.Wallin, StefanFaller, PeterMaraia, RichardWang, QinhongFang, XianjunMarkus, HenkeWeldon, SineadFaraggi, EshelMeng, QinWen, FeiFariselli, PieroMerrill, Alfred H.Winterhalter, MathiasFerreon, Allan Chris M.Meyer Zu Heringdorf, DagmarWitwer, KennethFerry, James G.Montclare, JinWoodley, John M.Fraaije, MarcoMueller, MichaelXu, JinboFukuhara, ShigetomoMulholland, Adrian J.Yamaguchi, YoshikiGago, FedericoMurate, TakashiYang, Chaoyong JamesGarcía-Fruitós, ElenaNagiec, Marek M.Yin, ZhaojunGerard, NuovoNakagawa, ShinichiYu, Michael S.Gomes, ClaudioNatalello, AntoninoYura, KeiGotor-Fernández, VicenteNatarajan, ViswanathanZhang, XunGotor, VicenteNevalainen, HelenaZou, Wen-QuanGreulich, KarinNishihara, Shoko


